# Household food insecurity in South Africa from 1999 to 2021: a metrics perspective

**DOI:** 10.1017/S1368980023001878

**Published:** 2023-11

**Authors:** Louise van den Berg, Corinna May Walsh

**Affiliations:** Department of Nutrition and Dietetics, University of the Free State, Bloemfontein 9300, South Africa

**Keywords:** Food security, Hunger, South Africa, Metrics, Systematic review

## Abstract

**Objective:**

To review and synthesize studies on household food security in South Africa.

**Design:**

Systematic mapping review of metrics (methodological review).

**Setting:**

Electronic databases, including EBSCOHost, Scopus and Web of Science, were searched for studies and reports on household food security in South Africa, reporting household food security published between 1999 and 2021. Searching, selecting and reporting were performed according to the PRISMA (Preferred Reporting Items for Systematic reviews and Meta-Analyses) statement.

**Participants:**

South African households

**Results:**

Forty-eight articles reporting on six national surveys (one repeated annually since 2002) and forty sub-national studies meeting the inclusion criteria were selected. Various metrics, with different recall periods and ways of categorizing food security levels, were identified. Surveys that used similar metrics showed that the percentage of South African households that have experienced food insecurity and hunger has decreased over the review period yet remains concerning. However, the multitude of metrics used to assess the different components and levels of food security limits the comparability of the results to evaluate the scope and scale of the problem.

**Conclusions:**

There is growing support for developing multi-variable approaches for food security research in sub-Saharan Africa. Future research should focus on finding the most appropriate combination of complementary metrics that would allow comparable data while holistically capturing food security and providing insight into the causes and consequences.

In pursuit of the UN Sustainable Development Goal to end hunger, monitoring the prevalence of food insecurity and identifying and studying the underlying drivers and consequences are vital to informing policy, strategies and programs^(1,2)^. The concept of food security was first defined in 1974. Since then, it has evolved from primarily focusing on food availability to being defined as ‘*physical, social, and economic access to sufficient, safe and nutritious food to meet dietary needs and food preferences for an active and healthy life by all people at all times*’^(3,4)^. This widely accepted FAO definition recognizes four dimensions of food security: food availability, access, utilization and stability^(4,5)^. Since the 1970s, food security has become differentiated not just at the global, regional and community levels, but eventually also at the household and individual levels, and revisions of the definition have come to include concepts of chronic and transitional food insecurity and, recently, human rights and ethics^(5)^. The modern concept of food security is thus a complex, non-material construct for which no single objective benchmark exists^(4,6,7)^. Many metrics have been developed to measure food security at different levels, but evidence shows that they may not all assess the same construct. Rather, each focuses on one or more of the four dimensions^(4,6,7)^. There is growing recognition that no single existing metric fully captures the intricacies of food security nor accounts for all determinants and sub-domains of food security in each context where it is applied^(4,6–10)^. Moreover, the evidence for validity and reliability of some metrics is not always clear^(10)^. These shortcomings complicate the measurement and interpretation of the role of food security at the household and individual level^(2,4)^ as drivers of malnutrition in countries like South Africa, where the prevalence of malnutrition in all its forms remains high^(11)^. The 2022 Global Nutrition Report notes that 21·4 % of children under 5 years of age are stunted, while 3·4 % are wasted and 11·6 % are overweight. In adults, 42·9 % of women and 18·2 % of men are classified as obese^(12)^.

A distinction is made between direct and indirect metrics to measure food access at the household level^(6)^. While indirect metrics rely on ‘second-generation’ indicators like household income and expenditure^(6,13)^, direct metrics use ‘third-generation’ indicators based on the paradigm that food insecurity is a quantifiable experience that can be described and analyzed^(14)^. Metrics were thus developed to reflect experiences related to household-level food access at different levels of food security. This development was based on research findings in the early 1990s that women, as primary caregivers in their households, see hunger as a ‘managed process’ and develop coping mechanisms that protect the children, often at the cost of their own nutrition, causing women and children in a household to experience different components of hunger at different times and to different degrees^(15,16)^. The experience-based metrics measure four constructs on the household and individual levels^(10,14–16)^. The first is a quantitative aspect of insufficient food indicated by food depletion in the household and perceived insufficient intake by individuals^(10,15)^. The second construct is a qualitative aspect that encompasses types and diversity of food indicated by perceived unsuitable food acquired by the household and nutritional inadequacy for the individuals^(10,15)^. Food quality is generally affected at the individual level before quantity^(6,13)^. The third construct is a psychological element as food insecurity, characterized by anxiety in the household over whether the food budget and amount and types of food available in the home would be sufficient to meet basic needs, and emotions of deprivation or limited choice for individuals^(10,15)^. These elements cause households to devise coping mechanisms to manage the situation^(15)^. The fourth is a social or normative aspect by which individuals in the household evaluate their (and their children’s) food situation in relation to generally accepted social norms, such as eating three meals a day or the household being able to purchase food without resorting to socially unacceptable behavior such as begging, relying on charity, scavenging or stealing food^(10,14–16)^. Food security is thus viewed as a spectrum of experiences ranging from starving to complete food security, which is described as a situation in which all the FAO (1996) criteria for food security are met, and there is no concern about future food supply, availability, and affordability to meet these criteria^(2)^. Experience-based metrics include the Community Childhood Hunger Identification Project (CCHIP) index, the US Household Food Security Survey Module (HFSSM), the Household Food Insecurity Assessment Score (HFIAS), the Household Hunger Scale (HHS), and the Food Insecurity Experience Score (FIES) and provide an assessment of food security that is directly related to the widely accepted FAO definition and have been validated and proven reliable across countries^(10,14–16)^. It could be argued that so-called consumption-based metrics, like the Household Dietary Diversity Score (HDDS) and the Food Consumption Score (FCS), are also more direct measures of food security^(10)^. However, concern has been raised that, although they are useful in combination with other metrics, they lack a clear model linking them to food security, which has prevented establishing their validity as food access metrics at the household level^(10)^. Despite the conceptual differences between metrics to assess household food security, users often apply them interchangeably^(2,10)^.

South Africa is a low and middle-income country (LMIC) with nine provinces, covering 1 219 090 km and 60,14 million people in 2021^(17)^. At the national level, South Africa is considered food-secure^(18)^, but there is widespread agreement that household food insecurity remains a serious problem^(19–22)^, emphasizing the critical need for differentiating the determinants. Several reviews had provided a comprehensive overview of household food security among adult South Africans since 1999 when the first national food security survey was conducted as part of the National Food Consumption Survey (NFCS)^(19–22)^. However, not captured in previous reviews are the 2019 and 2020 General Household Surveys (GHS)^(23)^ and the 2020/2021 National Income Dynamics Study’s Coronavirus Rapid Mobile Survey (NIDS-CRAM)^(24)^. Furthermore, given the current debate that diverse metrics may complicate the interpretation of surveys, also in the South African context^(4,12)^, this review aimed to provide an updated overview of the prevalence of household food security recorded in South African national and sub-national studies from 1999 to 2021, with emphasis on the different metrics used and the potential implications for defining the prevalence and determinants of household food security in the country.

## Methods

### Electronic literature search strategy

An electronic search of the following databases was performed to identify studies and reports on food security published from 1999 until the end of 2021: EBSCOHost (Academic Search Ultimate, Africa-Wide Information, CAB Abstracts, CINAHL with Full Text, GreenFILE, Health Source – Consumer Edition, Health Source: Nursing/Academic Edition, APA PsycArticles, APA PsycInfo, Sociology Source Ultimate, MEDLINE, MasterFILE Premier); Scopus; and Web of Science. In addition, the reports of national surveys that have been undertaken since 1999 were downloaded, and additional relevant studies in reference lists of retrieved articles were also included. The overarching review was related to an assessment of nutritional status, including studies on food security and hunger, using the following search terms: South Africa* (household* or national*) and (food* or nutrition*) and (secur* or insecur* or adequa* or access* or availab* or povert*) or hunger) (food* or nutrition* or secur* or insecur* or adequa* or access* or availab* or povert* or hunger). It is possible that, despite all these efforts, there may be publications and reports with valuable information on the food security of South Africans that were not identified.

### Inclusion and exclusion criteria

Reports of national surveys undertaken since 1999 and sub-national studies with data collection between 1999 and 2021 published in English in peer-reviewed journals as original articles on household food security carried out in South Africa were included in the current review. Review articles, unpublished studies or studies reported only as abstracts, studies undertaken in participants that were pregnant or lactating, had a diagnosis such as those that were HIV-infected, tuberculosis or a chronic condition (e.g. CHD, diabetes, cancer or disabled), and hospital-based studies were excluded from the review. National surveys using indirect food security metrics like the Income and Expenditure Survey (IES), Labour Force Survey (LFS) and Community Surveys Stats were excluded. Studies using dietary diversity metrics were only included if the stated intended use was to measure food security but were excluded if the primary objective was to use dietary diversity as a proxy of micronutrient intake.

### Data extraction

A systematic mapping review of metrics (methodological review)^(25)^ to assess household food security in South Africa over the reference period was conducted using the PRISMA (Preferred Reporting Items for Systematic reviews and Meta Analyses) recommendations of 2015^(26)^. All the study titles and relevant abstracts were read by the two authors, who agreed on the eligibility of studies for inclusion in the review. All duplicate articles were removed using Mendeley software version1.19.5/2019 (Elsevier, London). Several articles were removed after reading the title and the abstract. The remaining full-text articles were read to identify studies that met the inclusion/exclusion criteria. Studies were categorized according to the year of data collection, site (province and specific location), geographic area (rural or urban), population and sampling (gender and ethnicity of participants, and sample size). The descriptive data per variable of interest were extracted from the publications for presentation in the tables, while categorical variables were described by the percentage of subjects with values in the different categories.

## Results

### Study selection

**Fig. 1:**
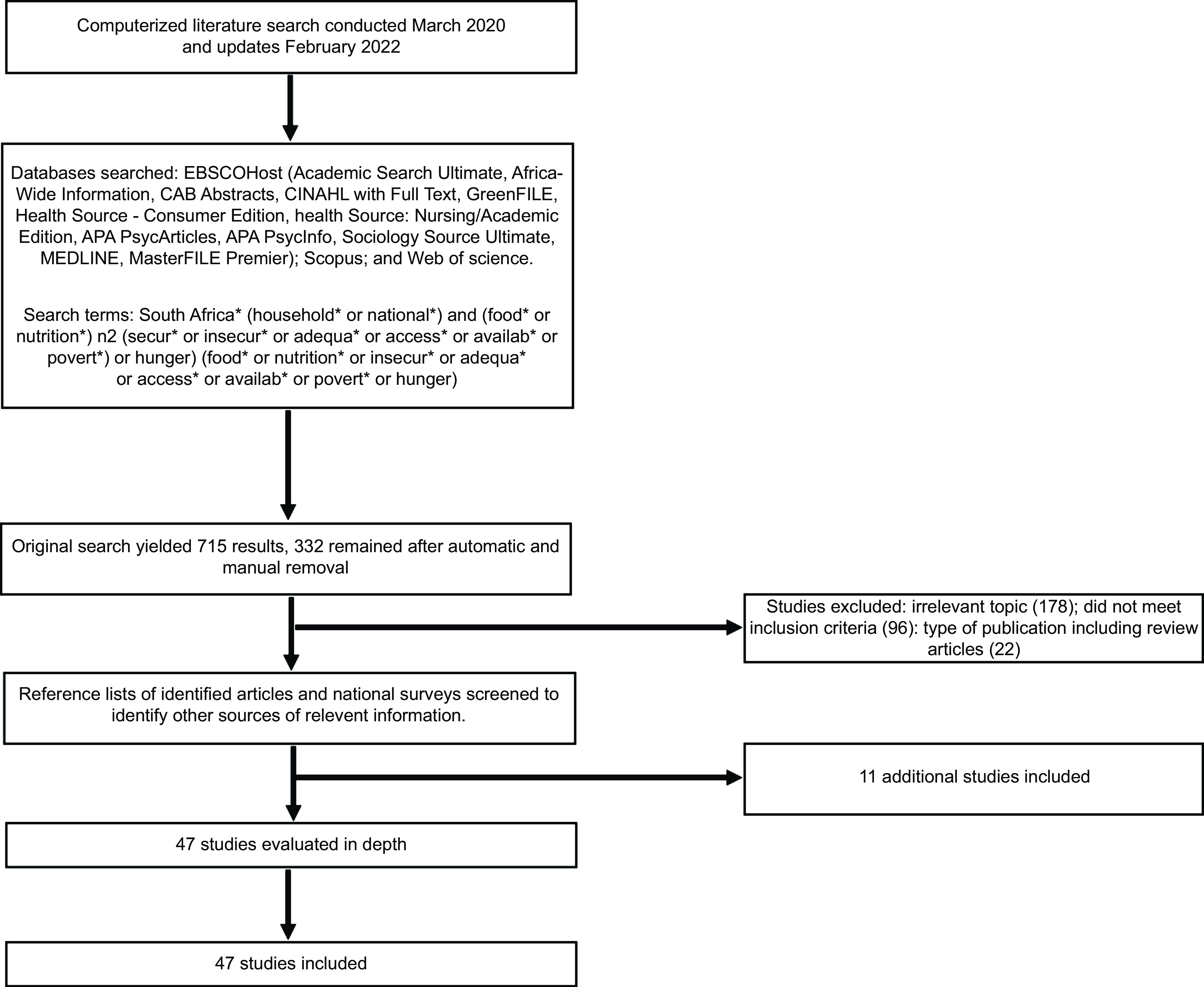
Flow chart representing the search, screening and selection of studies

We identified a total of 715 original articles in six databases. After automatic system deletion of the duplicates and further manual removal of the remaining duplicates, 332 were retained. A title and abstract-based selection resulted in the exclusion of 178 articles that were irrelevant and 118 that did not meet the inclusion criteria (twenty-two of which reviewed articles). After reading the full text of the remaining thirty-six articles, eleven additional articles and reports from their reference lists were included. Thus, forty-seven articles reporting on six national surveys and forty sub-national studies meeting the inclusion criteria were selected. The representative schema of the research and the number of eligible studies are shown in Fig. 1.

### Metrics and prevalence of household food security in national surveys

The search parameters identified six nationally representative surveys (Table 1). Three of these, namely the 1999 National Food Consumption Survey (NFCS 1999), the National Food Consumption Survey – Fortification Baseline (NFCS-FB) and the South African National Health and Nutrition Examination Survey (SANHANES-1), used the eight-item CCHIP index to assess household food security with a recall period of 3 months^(33)^. The NFCS 1999 reported that nationally 52 % of households ‘experienced hunger’ (extreme food insecurity), while 23 % were ‘at risk of hunger’. More households in rural areas (62 %) than urban areas (42 %) reported experiencing hunger. Moreover, hunger was more prevalent in informal urban (61 %) and informal rural areas (66 %) compared to formal urban (37 %) and formal rural (48 %)^(29)^. The NFCS-FB, undertaken 6 years later^(30)^, found a similar national prevalence of household hunger as the NFCS 1999, with 51·6 % experiencing hunger and 28·2 % at risk of hunger^(30)^. However, the percentage of participants that experienced hunger in informal rural areas had increased from 48 %^(29)^ to 58 %^(30)^.

**Table 1:**
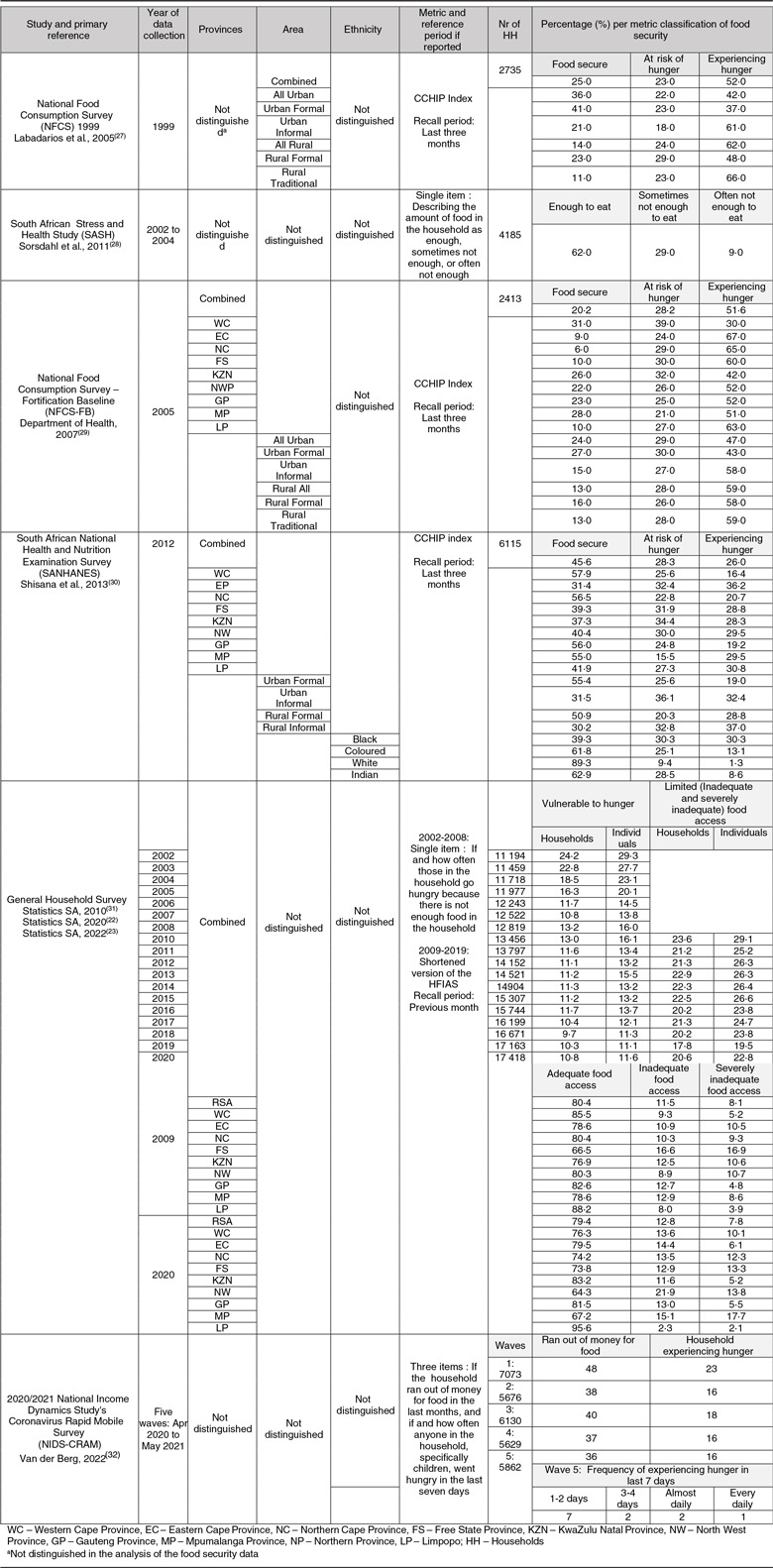
National surveys of household food security status of South African adults (1999–2021)

The SANHANES-1 followed in 2012^(34)^; by this time, the national prevalence of households experiencing hunger had decreased to 26·0 %, with 28·3% still at risk of hunger. The prevalence of food insecurity had dropped in formal and informal urban areas and formal rural areas but had increased to 37% in informal rural areas. The prevalence of hunger remained higher in rural compared to urban areas and in informal areas compared to formal areas^(34)^. Data were also analysed according to ethnicity, showing that the highest prevalence of hunger occurred in South African households of Black Africans (30·3%) and those of mixed ethnic origin (referred to as Coloureds by Statistics South Africa) (13·1%), followed by Indians (8·6%), while only 1·3% of White households experienced hunger. A comparison between the provincial data from the NFCS-FB (2005) and SANHANES (2012) shows that hunger declined in all nine provinces over the 7 years between the surveys, which agrees with the decline in multidimensional poverty in the country over the same time frame^(28)^. Hunger remained most prevalent in the Eastern Cape (2005: 67% *v*. 2012: 36·2%) and least prevalent in the Western Cape (2005: 30% *v*. 2012: 6·4%). A decline in food insecurity was most pronounced in the Northern Cape (2005: 65% *v*. 2012:20·7%), followed by Gauteng Province (2005: 52% *v*. 2012:19·2%).

Other nationally representative surveys that collected data on household food security included the South African Stress and Health Study (SASH), the South African GHS and, most recently, the 2020/2021 NIDS-CRAM. SASH collected data from 2002 to 2004 using a single question to assess food access (‘Which of the following describes the amount of food your household has to eat: enough to eat, sometimes not enough to eat, or often not enough to eat?’)^(35)^. The recall period is not reported but was presumably the last 12 months, as the other metrics in the survey used this reference period^(32)^. SASH reported that 38 % of households ‘sometimes’ or ‘often’ did not have ‘enough to eat’.

The GHS has been conducted annually since 2002. Until 2008, it included only one question to assess hunger (‘How often do adults and children go to bed hungry because there is not enough food in the household’). From 2009 onwards, a shortened version of the HFIAS was added to capture food access, with the last month as the recall period. The GHS single-question metric indicates that the percentage of households ‘vulnerable to hunger’ decreased from 24·2 % in 2002 to 11·8 % in 2020. The HFIAS showed that the percentage of households that had ‘inadequate’ and ‘severely inadequate access’ (pooled for reporting purposes as ‘limited access’) to food decreased from 26·3 % in 2010 to 17·8 % in 2019 and then rose again to 20·6 % in 2020^(23)^. The GHS has tracked provincial food access since 2009. From 2009 to 2020, the prevalence of severely inadequate food access decreased in four provinces: KwaZulu Natal, Eastern Cape, Free State and Limpopo Provinces, but increased in the other five provinces, most markedly in Mpumalanga. Food security data and overlapping time points between the NFCS (1999), NFCS-FB (2005), SANHANES (2012) and the GHS data are summarised in Table 2.

**Table 2:** Comparison of reported food security prevalence (%) at overlapping time points in South African national surveys (1999–2021)

Overlapping time points	CCHIP index			GHS single-item metric^(22,23)^		GHS shorted HFIAS^(22,23)^		SASH single-item metric^(28)^	
		Being hungry		Vulnerability to hunger		Limited food access (inadequate and severely inadequate access combined)		Having enough to eat	
Year	Survey	At risk of hunger	Experiencing hunger	Households vulnerable to hunger	Individuals vulnerable to hunger	Households	Individuals	Sometimes enough to eat	Not enough to eat
2002	NFCS^(27)^	23·0	52·0	24·2	29·3	–	–	29	9
2005	NFCS-FB^(29)^	28·2	51·6	16·3	20·1	–	–	–	–
2012	SANHANES^(30)^	28·3	26·0	11·1	13·2	23·1	26·3	–	–

CCHIP, Community Childhood Hunger Identification Project; GHS, General Household Survey; HFIAS, Household Food Insecurity Assessment Score; SASH, South African Stress and Health Study; NFCS, National Food Consumption Survey; NFCS-FB, National Food Consumption Survey – Fortification Baseline; RSA, Republic of South Africa; SANHANES, South African National Health and Nutrition Examination Survey.

Finally, the NIDS-CRAM was conducted in five waves of data collection from March 2020, when a national lockdown was mandated in response to the international coronavirus pandemic, until May 2021 to assess the impact of the pandemic on household food security. The NIDS-CRAM used three items, one related to food access, asking if the household ran out of money for food in the last month, and two asking if anyone in the household, including children, went hungry in the last 7 days and how often, reporting the results in these terms instead of using scoring scales^(36)^. The survey found that food access improved over the five waves, from 48 % of households running out of money for food in March 2020 to 36 % in May 2021. The number of individuals that went hungry initially dropped from the first wave (23 %) to the second wave (16 %) but then stabilized at that level. In wave 5, with data collection in April/May 2021, 3 % of adults reported experiencing hunger daily or almost daily in the last week.

### Metrics and prevalence of household food security in sub-national surveys

Thirty six sub-national studies fit the inclusion criteria (excluding four studies focused on students in higher education). These studies (Table 3) used a variety of metrics: HSFIAS (*n* 16)^(44,46,48–50,52,55,56,59–61,63,66,67,69,70)^, CCHIP (*n* 2)^(41,58)^; Cornell Hunger Scale (*n* 2)^(42,43,71)^; HHS (*n* 3)^(2,40,62)^; and Household Food Insecurity Access Prevalence (HFIAP) (*n* 1)^(69)^; single-item (*n* 3)^(45,47,57)^ or two-item metrics (*n* 1)^(38)^; Food-Coping Strategy Index (*n* 1)^(65)^; Coping Strategies Index (CSI) (*n* 2)^(2,56)^; FCS (*n* 3)^(2,54,65)^; HDDS (*n* 1)^(54)^; Modified Complex Access to Food (mCAF) score (*n* 1)^(2)^; Months of Adequate Household Food Provisioning (MAHFP) (*n* 2)^(56,67)^; months of food shortages (*n* 1)^(63)^; food access based on a composite econometric model (*n* 1)^(39)^; Household Food Intake Index developed from principal component analysis (*n* 1)^(53)^; low energy availability (*n* 1)^(67)^; household food accessibility based on per capita energy intakes (*n* 3)^(53,64)^, including the Food Poverty Index (FPI) (*n* 1)^(67)^; and household food accessibility based on household food expenditure (*n* 2)^(68,72)^. Recall periods varied according to the metric and included 1 year, 30 d, 1 month, 7 d, 5 d and 24 h. Prevalence of food security was reported using a wide variety of scoring systems reported as mean scores or in categories using an array of terminology.

**Table 3:**
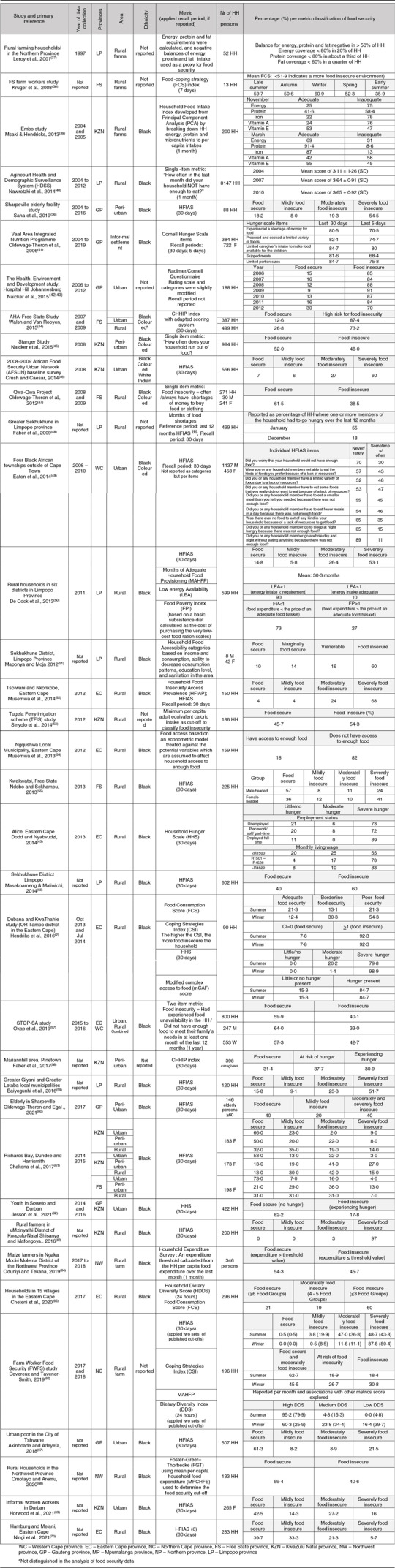
Published sub-national studies of household food security status of South African adults (1999–2021)

As summarised in Table 3, most of the included sub-national studies were conducted in rural areas (*n* 27), while thirteen studies recorded data in urban areas (five in peri-urban areas). The prevalence of severe food security ranged from 3 % to 97 % depending on the metric used and was consistently higher in rural areas compared to urban areas across studies and within studies that used the same metrics. Nine studies collected data in KwaZulu Natal province, seven in the Eastern Cape province, six in Gauteng province, seven in Limpopo province, five in the Free State province, two in Northwest province, one in the Northern Cape Province, one in the Western Cape province and none in Mpumalanga province. Studies included mostly only Black participants, with five studies including Coloured participants and only one study also including White and Indian participants. Six studies did not report ethnicity. Food security was almost exclusively reported per household, with women mostly being the interviewees. None of the studies reported food security per individual in the household, and only one focused specifically on elderly individuals, finding that 54·5 % of participants ≥ 60 years (Black, from a peri-urban area in Gauteng province) were severely food-insecure, and none had high food security^(50)^.

Four studies reported on the prevalence of food security among South African university students (Table 4), adapting the wording of the metrics to apply to students. However, most of these manuscripts did not indicate how this was done or which reference periods were used. In 2012, using the HFIAS, 12·5 % of students at the University of KwaZulu Natal (UKZN) receiving government aid to support their studies were classified as food-insecure and 53·1 % at risk of food insecurity^(73)^. In 2013, a single-item metric and the eight-item HFSSM were used to collect data on a representative sample of all students registered at the University of the Free State (UFS). The reference period was defined as the academic term while studying at the university from the beginning of the academic year to exclude university breaks when students are not studying from home and might find themselves in a different food situation. The single-item metric (‘In the last 12 months, during the academic term, were there any times that you ran out of food and couldn’t afford to buy any more?’) classified 64·5 % of students as food-insecure. For this survey, the classification system for the HFSSM was slightly adapted from the published metric, and it was reported that 24 % of respondents had marginal or low food security, and 60 % had very low food security^(75)^. Another two surveys at the University of the Witwatersrand (WITS) in 2018^(74)^ and 2019^(76)^ used the HFIAS to assess food insecurity among students. The first^(74)^ reported only the HHS, finding that 1 % of students were experiencing severe hunger and 6 % were experiencing moderate hunger. The second was conducted among a representative sample of first-year students who were enrolled in 2019^(76)^. According to the HFIAS, 73 % of respondents in this survey were classified as food-insecure; 38 % were severely so. According to the HHS, 18 % were moderately hungry and 5 % were severely hungry. An ‘integrated’ scoring was also reported, showing that 59 % were food-insecure without hunger and 23 % were food-insecure with severe hunger.

**Table 4:** Published surveys of food security status of South African adult students in higher education (1997–2021)

Study and primary reference	Data collection	Nr of students	Metric	Percentage (%) per metric classification of food security						
University of KwaZulu Natal Kassier and Veldman., 2013^(72)^	2012	269	Household Food Insecurity Access Scale (HFIAS)	Food-secure		At risk of food insecurity			Food-insecure	
				34·4		53·1			12·5	
University of the Free State Van den Berg and Raubenheimer, 2015^(73,74)^	2013	1413	Single-item measure: ‘In the last 12 months, during the academic term, were there any times that you ran out of food and couldn’t afford to buy any more?’	Food-secure				Food-insecure		
				35·5				64·5		
			8-item Household Food Security Survey Module (HFSSM) (scoring adapted)	Food-secure (high and marginal food security combined)		Food-insecure without hunger (low food security)			Food-insecure with hunger (very low food security)	
				15·4		24·6			60·0	
University of the Witwatersrand Rudolph et al.^(75)^	2018	387	HFIAS Household hunger subscale (HHS) (reported only)	Little or no hunger		Moderate hunger			Severe hunger	
				97		6			1	
University of the Witwatersrand Wagner et al.^(74)^	2019	1612	HFIAS	Food-secure	Mildly food-insecure		Moderately food-insecure			Severely food-insecure
				27	11		24			38
			HHS	Little or no hunger		Moderate hunger			Severe hunger	
				77		18			5	
			Integrated interpretation	Food-secure		Food-insecure without hunger			Food-insecure with hunger	
				27		59			23	

## Discussion

Assessment of food security depends to a large extent on the methodology employed. This systematic review included six national surveys (one repeated annually since 2002) and thirty-six sub-national studies reporting household food security conducted from 1999 to 2001. The wide variety of metrics and different ways of reporting the findings limit the comparability of the results to measure the scope and scale of the problem.

Regularly conducting nationally representative surveys is important for tracking changes in food security over time to guide policies, programs and strategies. Valid and comparable metrics are required to compare data over time and across populations. However, food security as a multidimensional construct is difficult to capture holistically in a single metric. Experienced-based multi-item metrics are considered more valid measures of food security than consumption-based metrics^(10)^ and are extensively used to track and compare global, regional and national food security. The HFSSM, based on items identified by Radimer et al. in the 1990s^(15,16)^, has been used to track food insecurity in the USA since 1995 and Canada since 2004. Subsequently, based on the items of the HFSSM, the HFIAS was developed for tracking food access in low and middle-income countries^(6)^. More recently, the FAO developed the FIES to establish an indicator for global monitoring of food insecurity that has been applied across countries and cultures since 2014^(77,78)^. Of the nationally representative surveys that measured food security in South Africa since 1999, three used the CCHIP index, namely the NFCS of 1999^(29)^, the NFCS-FB of 2005^(30)^ and SANHANES-1 of 2012^(34)^. The GHS started collecting annual food security data in 2002. Up to 2008, only a single-item metric was used that only asked whether the household ran out of money for food and if and how frequently they experienced hunger. From 2009 onward, a shortened version of the HFIAS was added. When the findings of the national surveys at overlapping time points (2002, 2005 and 2012) are compared (Table 2), it becomes clear that the reported prevalence, expressed as the percentage of participants that represent different levels of food insecurity, is hardly comparable, suggesting that the metrics used were not measuring the same construct of food security.

Moreover, the terminology used to describe the categories with each metric is difficult to compare. Surveys using the CCHIP reported the prevalence of food security as the percentage ‘at risk of hunger’ and ‘experiencing hunger’. The single-item measure used by the GHS reported the prevalence of those ‘vulnerable to hunger’ (the shortened version of the HSFIA used in the GHS since 2009) reported the prevalence of those with ‘limited food access’ (which combines those with ‘inadequate’ and ‘severely inadequate’ access). The single-item measure used in SASH reported the prevalence of those who ‘do not have enough to eat’. Jones et al. (2013) pointed out that many disciplines, including agriculture, economics, nutrition, public policy, anthropology and sociology, engage with food security, each contributing its jargon, so that terminology has become confusing and terms that represent different constructs are often used interchangeably^(6)^.

Nevertheless, these three metrics showed the same trend of decreasing levels of food insecurity over the reference period, even though they may have measured slightly different food security constructs. However, this poses a problem for comparing the prevalence of food insecurity between studies using different metrics.

The difficulties incurred by the diversity of metrics and the diverse classification systems and terminology used to classify household food security are even more apparent in the sub-national surveys summarised in Table 3. Six different previously validated experienced-based metrics, namely HSFIAS, CCHIP, Cornell Hunger Scale, HHS and HFIAP, were used, with some studies adapting the scoring systems and/or reporting the prevalence of food security by combining the categories that represent the level of food security in the household, in different ways. Four studies used other single- or two-item metrics based only on the quantitative component usually represented by the first item of the other experience-based metrics.

Three studies used indexes based on the frequency and severity of the household’s coping mechanisms in the face of food insecurity^(2,56,65)^. These metrics were not intentionally moulded on the experienced-based metrics but attempt to capture the behaviour of individuals faced with ‘uncertainty, irreversibilities, and binding constraints on choice’^(4)^, thus introducing the element of ‘perceived vulnerability’^(4)^. These metrics cannot classify households along the food security continuum, and as coping strategies differ according to the study’s specific context, community engagement is necessary to establish severity levels for each strategy. While these metrics are unsuitable for comparative national surveys, they render important information for designing appropriate intervention programs^(2)^. Four studies used direct metrics of household food security that can be classified as consumption-based metrics, namely the FCS^(2,54,65)^ and the HDDS^(39)^, which attempt to define a concept of food consumption that would reflect both quantity and quality^(7,10)^. Variety is a key element of high-quality diets. However, while dietary diversity scores may be significantly associated with food insecurity in the South African context^(63)^, it is not clear to what extent dietary diversity consistently reflects differences in the food security status of households or individuals^(10)^. Thus, it is recommended that dietary diversity scores should be used in combination with other food security measures^(2,6,7)^. Several other metrics used in the sub-national studies focus on how long the household had experienced limited access to food, including the mCAF score^(2)^, MAHFP^(56,67)^ and months of food shortages^(63)^. Lastly, several metrics focused on food access based on meeting per capita energy requirements^(39,53,64,67)^ or on how much a household can spend on food^(68,72)^. These metrics measure very different constructs compared to the HSFIAS, CHHIP, Cornell Hunger Scale and HHS.

Notably, even the experience-based metrics, which were designed not only to capture the quantitative aspects of food access but also the psychological and normative aspects embodied in the FAO definition of food security, have limitations when considered in the context of the various national surveys and sub-national studies included in this review. The interviewee in almost all of the included studies portraying household food security research over the last two decades was a single female representing the household. Hendriks et al.^(2)^ note that ‘the experience of hunger is not universal and perception of what constitutes being hungry differs according to context, culture, and experience’. Concerns have been raised that the experience-based metrics (used in all of the national surveys and most of the sub-national studies included in the current review) were developed based on research by Radimer et al. in the 1990s^(15,16)^ on the perceptions of women in the household. Radimer et al. (1992)^(15,16)^ pointed out that the metric was standardized on women and that application to men and the elderly would need further investigation, but no progress has been made in this regard. A case in point is the measurement of food security among students in higher education, which has become a global issue. The most widely used metric for assessing food insecurity among students globally is the HFSSM. However, cognitive interviewing with US students recently found that they interpreted key terms, such as ‘money for more’, ‘balanced meals’ and ‘real hunger’, differently from theoretical dimensions^(79)^. South African studies of student food insecurity have used the HFSSM and the HFIAS (Table 4), but as these two metrics share similar terminology, they would likely incur the same problems when assessing students.

A household is considered food-insecure when it contains one or more food-insecure individuals. At the same time, though, various authors argue that a single individual respondent cannot accurately represent the experience of others in their household in an interview^(4,8)^. Coates^(4)^ argues that while children’s food security is related to that of adults in the same household, it depends on the child’s age. Subsequently, it seems that separately measuring children’s and adults’ food security is better than one measure that tries to represent both, as current experience-based metrics were designed to do. El-Rhomri and Domínguez-Serrano^(8)^ note that household members do not always pool their resources equitably, for example, due to gender dynamics and gender power imbalance. Furthermore, members may share responsibilities to provide food, which can change according to circumstances. Members of a household may also obtain food from various sources not figured into the assessment. Similarly, coping strategies often used to assess food security (as in the CSI) vary between regions, communities, social classes, ethnic groups, households, gender, age and seasons^(8)^. These nuances are not necessarily captured by approaches that only interview one person representing the household.

The geographical location of households raises another concern concerning the metrics used. This systematic review highlights that food insecurity over the last two decades has decreased in South Africa but remains high in rural areas. The lack of natural resources to sustain agricultural livelihoods leading to the abandonment of own food production and prevailing gender inequality have been identified as major drivers of high food insecurity in rural South Africa^(69,80)^. Most included sub-national studies focused on rural areas, with less emphasis on urban areas. However, the urban population in sub-Saharan Africa is projected to increase from 376 million in 2015 to over 1·25 billion people by 2050^(81)^. The effect of this rapid urbanization on food security needs to become a research priority^(9)^, also in the context of the nutrition transition and the impact on malnutrition. Haysom and Tawodzera (2018)^(27)^ point out that food security metrics currently used to measure household-level food security in urban areas may be more appropriate to the rural contexts where they have been extensively used. They state that these metrics ‘may not shed light on the broader urban food system, including infrastructure challenges, travel, food safety, and market governance’.

The recall period is another factor that varied much between the metrics used in the studies included in this review. The periods varied from 24 h to 7 d, to 30 d (1 month), to 3 months to a year. Longer recall periods reflect chronic food security, while short periods reflect short-term vulnerability. In the context of studies like the NIDS-CRAM, a very short recall period was valid based on the purpose of the survey to assess the impact of the COVID-19 pandemic and the impact of emergency government assistance and other interventions on household food security in the country. However, most studies included in this review did not indicate that they had specifically considered the recall period. In the context of university students, for example, using a reference period of 12 months or indeed any period that spans recesses where students vacate the university residences and student housing that they occupy during the academic term to return to possibly different household food situations may complicate the interpretation and comparability of food security results in this context. Therefore, considering the purpose of planned food security surveys is vital when deciding on the recall period. Jones et al. (2013)^(6)^ also emphasized the importance of training fieldworkers to communicate to survey participants the same conceptual understanding of recall periods to temper recall bias. These considerations will only become more important considering a rising incidence of food security shocks linked to catastrophic climate change events, economic upheaval, civil unrest and war, amongst others, that have immediate and long-term effects on household food security, disproportionally affecting the poor.

There is growing recognition that we can no longer rely on a single metric when conducting food security research in the sub-Saharan African context, but that multi-variable approaches, drawing on the toolboxes of multiple disciplines, are vital^(4,6–10)^. Among the studies that met the inclusion criteria of this review, only two sub-national studies used multiple complementary food security metrics. The most appropriate combination of metrics and recall periods needs further research^(2,6,10,61)^. An in-depth analysis of the levels and components for which the available metrics are validated is vital^(10)^. Furthermore, few studies have assessed whether results obtained with common household food security indicators converge^(2,9)^. Evidence suggests that a panel of metrics chosen to assess food insecurity should also include metrics that assess the causes and consequences of food insecurity^(2)^. Since food security metrics are not sensitive enough to identify those who most need support, anthropometric measurements should be included^(2,4,31)^. Furthermore, dietary diversity data should be included because, while the experience of hunger reflects the presence and frequency of deprivation, it does not provide information on the quality of the diet^(2,6)^. Notably, none of the current food security metrics *per se* provides insight into the causes of food insecurity^(2)^. Thus, food security studies should be designed also to measure variables related to the determinants of food insecurity to inform intervention.

The following limitations are acknowledged: The quality of data collected in all studies included in a review of this nature cannot be assured. Although all identified studies were included in the current review, it may be argued that studies with very small sample sizes or inappropriate assessment methods should have been excluded. The authors decided to include them to provide a holistic picture of the work done over the review period, but their data have not been taken into account in the conclusions.

### Conclusion

Although the current review suggests that the percentage of South African adults that have experienced food insecurity and hunger has decreased over the review period, the multitude of metrics used to assess the different components and levels of food security make it difficult to draw definitive conclusions. There is growing support for developing multi-variable approaches for food security research in sub-Saharan Africa. Future research should focus on finding the most appropriate combination of complementary metrics that would allow comparable data while holistically capturing food security and giving insight into the causes and consequences. Many South Africans still experience food insecurity and hunger regardless of the metrics used.
